# Multi-outcome prognostic modelling for older adults after trauma: development and validation of the Older Trauma Outcome Predictor (OTOP) model

**DOI:** 10.1093/bjsopen/zrag084

**Published:** 2026-07-20

**Authors:** Mayura Iddagoda, Michelle Trevenen, Dieter G Weber, Christopher Etherton-Beer, Leon Flicker

**Affiliations:** School of Medicine, University of Western Australia, Perth, Western Australia, Australia; Perioperative and Aged Care Service, Royal Perth Hospital, Perth, Western Australia, Australia; School of Medicine, University of Western Australia, Perth, Western Australia, Australia; State Adult Major Trauma Service, Royal Perth Hospital, Perth, Western Australia, Australia; School of Surgery, University of Western Australia, Perth, Western Australia, Australia; School of Medicine, University of Western Australia, Perth, Western Australia, Australia; Perioperative and Aged Care Service, Royal Perth Hospital, Perth, Western Australia, Australia; School of Medicine, University of Western Australia, Perth, Western Australia, Australia; Perioperative and Aged Care Service, Royal Perth Hospital, Perth, Western Australia, Australia

## Abstract

**Background:**

Older adults account for a rising proportion of trauma admissions, yet most prognostic tools for this group predict mortality alone. A validated multi-outcome model could support early risk stratification and optimize trauma care planning. This study developed and validated the Older Trauma Outcome Prediction (OTOP) model, the first tool designed to estimate multiple inpatient outcomes in older trauma patients.

**Methods:**

This study used Western Australia Trauma Registry linked data on patients aged ≥ 50 years admitted after external injury (2000–2020). Predictor variables, identified from a systematic review, included demographic factors, co-morbidities, injury characteristics, physiological measurements, and resuscitation indicators. The cohort was randomly divided into training (80%) and validation (20%) sets. Ridge logistic regression was used for binary outcomes (in-hospital mortality, surgery, intensive care unit (ICU) admission, medical complications), multinomial logistic regression was used for discharge destination, and quasi-Poisson regression was used for hospital length of stay. Model performance was assessed using the area under the curve (AUC), calibration, and error metrics.

**Results:**

This study analysed 105 972 trauma admissions aged ≥ 50 years between 2000 and 2020 (84 778 patients in the training cohort, 21 194 in the validation cohort). The mean age of all patients was 74.0 years, and 45.3% were men. Mortality prediction showed excellent discrimination (AUC 0.95), outperforming the Geriatric Trauma Outcome Score (AUC 0.83). The surgery and ICU admission models performed well (AUC 0.97 and 0.96, respectively). Length-of-hospital-stay prediction demonstrated moderate accuracy (mean absolute error 4.9 days), whereas prediction of medical complications was limited (AUC 0.60). Discharge destination was accurately classified into home, rehabilitation, residential care, or death.

**Conclusion:**

OTOP is the first validated multi-outcome prognostic model for older trauma patients, providing accurate and clinically relevant predictions to support early decision-making and optimize trauma system resource use.

## Teaser

This study developed and validated the Older Trauma Outcome Prediction (OTOP) model, the first prognostic tool designed to predict multiple inpatient outcomes in older trauma patients. Using data from more than 105 000 patients in the Western Australia Trauma Registry, OTOP accurately predicted mortality, surgical intervention, intensive care unit (ICU) admission, length of hospital stay, medical complications, and discharge destination. The model demonstrated excellent performance for mortality, surgery, and ICU admission and outperformed existing mortality-focused tools. OTOP provides a clinically useful framework for early risk stratification and supports more informed decision-making and resource planning for the care of older trauma patients.

## Introduction

The demographic profile of trauma patients is shifting, with an increase in the proportion of older individuals who present with multiple co-morbidities and frailty.^1,2^ This trend reflects global population ageing and has important implications for trauma care. Compared with younger patients, older people experience higher complication rates and worse outcomes, including prolonged rehabilitation, institutionalization, disability, and mortality^2–4^. Physiological changes associated with ageing, combined with frailty and chronic disease, further compromise surgical outcomes in this population^4^.

Several prognostic tools have been developed to predict mortality in older trauma patients, including the Trauma and Injury Severity Score^5^, the Geriatric Trauma Outcome Score (GTOS)^6^, the quick and full Elderly Mortality after Trauma scores^7^, and the Geriatric Trauma Mortality Score^8^. These models have demonstrated validity in estimating survival^9,10^. However, they do not predict other clinically significant outcomes, such as length of hospital stay (LOS), complications, surgical intervention, intensive care unit (ICU) admission, and discharge destination. The ability to anticipate such outcomes is essential for effective risk stratification and early optimization of care.

Predictors of non-mortality outcomes in older trauma patients are heterogeneous. A recent systematic review^11^ identified 43 factors associated with mortality, hospital stay, complications, rehabilitation requirements, and discharge to residential care, yet no comprehensive prognostic model has been developed. Addressing this gap requires evaluating multiple outcomes against relevant predictors to guide personalized care pathways.

The aim of this study was to establish a holistic prognostic model (Older Trauma Outcome Predictor) to predict multiple in-patient outcomes after major trauma in older adults. The model will integrate injury characteristics, physiological parameters, baseline co-morbidities, and resuscitation components, and is hypothesized to provide accurate risk stratification before surgical intervention, enabling targeted optimization and improved patient outcomes.

## Methods

### Data source

The Western Australia Trauma Registry is a statewide database established in 1994 that collates trauma-related information from the time of injury to discharge home or to rehabilitation^12^. For this study, data from Western Australia Trauma Registry were used to develop the Older Trauma Outcome Prediction (OTOP) model.

### Study population

Records were extracted from the registry for trauma admissions between 1 January 2000 and 31 December 2020. Patients were eligible for inclusion if they were aged ≥ 50 years and fulfilled the admission criteria for external injuries within the Western Australian health system. Patients who died at the scene of injury or in the emergency department were excluded.

### Predictor variables and outcomes

Candidate predictors were selected based on a systematic review^11^ and limited to variables available in the registry. These were broadly categorized into demographic characteristics, patient-related factors, injury characteristics, and admission measurements. Twenty-five predictors were chosen from 43 identified in the systematic review, informed by clinical relevance, trauma surgeon input, and international trauma reporting standards. Units for continuous variables and categories for categorical variables are presented in *Table 1*. Variables were intentionally retained in simple formats to minimize assumptions and enhance usability at the initial phase of clinical presentation. For example, co-morbidity burden was quantified as a straightforward count of documented conditions using routinely recorded diagnostic data from the Western Australia Trauma Registry and linked hospital data sets.

**Table 1:** Baseline characteristics and outcomes for the overall study population and for the training and validation groups separately

	Training group (*n* = 84 778)	Validation group (*n* = 21 194)	Overall (*n* = 105 972)
**Demographics**			
Age (years), mean(s.d.)	74.1(13.3)	74.0(13.4)	74.0(13.3)
Sex			
Female	46 359 (54.7%)	11 640 (54.9%)	57 999 (54.7%)
Male	38 419 (45.3%)	9554 (45.1%)	47 973 (45.3%)
Indigenous status			
Indigenous	1819 (2.1%)	429 (2.0%)	2248 (2..1%)
Non-Indigenous	82 945 (97.8%)	20 763 (98.0%)	103 708 (97.9%)
Missing	14 (0.0%)	2 (0.0%)	16 (0.0%)
Weight (kg,) mean(s.d.)	83.2(20.1)	82.3(20.0)	83.0(20.1)
Missing	78 894 (93.1%)	19 724 (93.1%)	98 618 (93.1%)
**Lifestyle variables**			
Had alcohol in past 12 h	3763 (4.4%)	953 (4.5%)	4716 (4.5%)
Smoker			
No	3599 (4.2%)	866 (4.1%)	4465 (4.2%)
Yes	1040 (1.2%)	264 (1.2%)	1304 (1.2%)
Missing	80 139 (94.5%)	20 064 (94.7%)	100 203 (94.6%)
Had illicit drugs in past 12 h	167 (0.2%)	44 (0.2%)	211 (0.2%)
**Clinical variables**			
No. of co-morbidities			
0	78 436 (92.5%)	19 652 (92.7%)	98 088 (92.6%)
1–2	2736 (3.2%)	665 (3.1%)	3401 (3.2%)
≥ 3	3606 (4.3%)	877 (4.1%)	4483 (4.2%)
Medication use (yes)	6004 (7.1%)	1471 (6.9%)	7475 (7.1%)
Workers’ compensation	1734 (2.0%)	440 (2.1%)	2174 (2.1%)
SBP (mmHg), mean(s.d.)	139(33)	139(33)	139(33)
Missing	78 107 (92.1%)	19 556 (92.3%)	97 663 (92.2%)
Pulse (beats/min), mean(s.d.)	82.0(21.3)	82.2(21.3)	82.0(21.3)
Missing	78 098 (92.1%)	19 551 (92.2%)	97 649 (92.1%)
Oxygen saturation (%), mean(s.d.)	94.0(12.2)	95.5(10.1)	94.3(11.9)
Missing	84 342 (99.5%)	21 101 (99.6%)	105 443 (99.5%)
Respiratory rate (breaths/min), mean(s.d.)	19.0(5.4)	18.8(5.4)	18.9(5.4)
Missing	78 462 (92.6%)	19 637 (92.7%)	98 099 (92.6%)
Temperature (°C), mean(s.d.)	36.4(1.1)	36.4(1.1)	36.4(1.1)
Missing	78 636 (92.8%)	19 680 (92.9%)	98 316 (92.8%)
Glasgow Coma Scale, mean(s.d.)	13.6(2.9)	13.6(2.9)	13.6(2.9)
Missing	78 979 (93.2%)	19 765 (93.3%)	98 744 (93.2%)
Haemoglobin (g/dl), mean(s.d.)	128(20.4)	128(20.8)	128(20.5)
Missing	79 976 (94.3%)	20 020 (94.5%)	99 996 (94.4%)
INR, mean(s.d.)	0.454(0.803)	0.424(0.689)	0.448(0.782)
Missing	73 839 (87.1%)	18 419 (86.9%)	92 258 (87.1%)
Base excess, mean(s.d.)	−4.14(6.41)	−4.95(6.27)	−4.31(6.38)
Missing	84 408 (99.6%)	21 100 (99.6%)	105 508 (99.6%)
Mechanism of injury			
Blunt	80 685 (95.2%)	20 120 (94.9%)	100 805 (95·1%)
Penetrating	4093 (4.8%)	1074 (5.1%)	5167 (4.9%)
ISS, mean(s.d.)	6.52(6.01)	6.51(5.99)	6.52(6.01)
Missing	18 (0.0%)	4 (0.0%)	22 (0.0%)
CT scan performed	6027 (7.1%)	1510 (7.1%)	7537 (7.1%)
Blood transfusion	762 (0.9%)	198 (0·9%)	960 (0.9%)
Intubation	1537 (1.8%)	355 (1.7%)	1892 (1.8%)
**Outcomes**			
In-hospital mortality	2320 (2.7%)	569 (2.7%)	2889 (2.7%)
Missing	36 (0.0%)	12 (0.1%)	48 (0.0%)
Surgery	3402 (4.0%)	829 (3.9%)	4231 (4.0%)
ICU admission	1727 (2.0%)	391 (1.8%)	2118 (2.0%)
Medication complication	3721 (4.4%)	917 (4.3%)	4638 (4.4%)
Missing	78 846 (93.0%)	19 729 (93.1%)	98 575 (93.0%)
Discharge destination			
Death	2320 (2.7%)	569 (2.7%)	2889 (2.7%)
Home	42 999 (50.7%)	10 715 (50.6%)	53 714 (50.7%)
Other	19 416 (22.9%)	4926 (23.2%)	24 342 (23.0%)
Rehabilitation	10 743 (12.7%)	2673 (12.6%)	13 416 (12.7%)
Residential care	9264 (10.9%)	2299 (10.8%)	11 563 (10.9%)
Missing	36 (0.0%)	12 (0.1%)	48 (0.0%)
LOS (days), median (i.q.r.)	5 (3–9)	5 (3–9)	5 (3–9)
Missing	3 (0.0%)	1 (0.0%)	4 (0.0%)

Values are *n* (%) unless otherwise stated. The groups are same sample and divided for model development and validation. Significance between group is not relevant in such situations. s.d., standard deviation; h, hours; SBP, systolic blood pressure; min, minutes; INR, international normalized ratio of prothrombin time; ISS, injury severity score; CT, computed tomography; ICU, intensive care unit; LOS, length of hospital stay; i.q.r., interquartile range.

The selected outcomes were chosen based on evidence from trauma and geriatric literature indicating that clinically meaningful endpoints in older adults extend beyond mortality alone. In addition to death, outcomes such as surgical intervention, ICU admission, medical complications, discharge destination, and LOS reflect injury severity, functional impact, loss of independence, and healthcare resource utilization. These domains were also identified in the preceding systematic review^11^ as key limitations of existing mortality-focused prognostic models.

### Handling of missing data

A substantial proportion of random missingness was present, particularly among admission measurements. Missing data for each variable are reported in *Table 1*. There was substantial missingness in source medical records relating mainly to clinical variables. To address this, multiple imputation by chained equations was employed, using 100 imputation sets, each consisting of 1000 iterations with a 30-iteration burn-in period. Auxiliary variables (trauma severity (major or minor), admission triage (resuscitation, emergency, urgent, semi-urgent), and admission to the state trauma unit (yes or no), all summarized in *Table S1* were included in the imputation model, because these correlated with at least one of the variables with missing data (correlation coefficient ≥ 0.4). The convergence of the imputation model was confirmed by inspecting the iteration plots.

### Model development

The data set was randomly divided into a training sample (80% of patients), whose data were used to develop the models, and a validation sample (20% of patients). Given the large number of predictors, ridge regression was applied to prevent overfitting; specifically, a logistic regression for binary outcomes (mortality, surgery, ICU admission, complications), a multinomial logistic regression for discharge destination, and a quasi-Poisson regression for LOS. Regression models generated outcome-specific coefficients for all predictors. Predicted probabilities were calculated using the combined linear predictor (the sum of all coefficients) rather than individual coefficients, ensuring that risk estimates reflect the joint contribution of all variables.

This study uses the sum of specificity and sensitivity to determine the cut-off point for each outcome, aiming to achieve balanced classification in the model. This method also enhances its usability in future artificial intelligence-driven model implementation.

For each binary outcome, the ridge regression was fitted across each of the 100 imputed training data sets. Predicted probabilities were calculated for each patient, and the optimal cut-off point was determined by maximizing the sum of sensitivity and specificity; a detailed explanation is provided in *Text S1* Coefficients and cut-off points were pooled across imputation data sets to generate the final model. The final model, including coefficients and cut-off points, was then applied to the 100 validation data sets. Model performance was assessed using the area under the receiver operating characteristic curve (AUC), accuracy, sensitivity, specificity, Brier score, and, for mortality, comparison with the GTOS, which is the simplest scoring system and is commonly used in clinical practice^6^. For discharge destination, multinomial logistic regression models were used to estimate the probabilities of five discharge categories: death, home, residential care, rehabilitation, and other. Sequential optimal cut-off points were applied in a stepwise manner, beginning with death and continuing through home, residential care, and rehabilitation (*Text S2E*). Patients not classified into any of these categories were assigned to the ‘other’ category. Pooled coefficients from imputed data sets defined the final prediction model. Validation performance was evaluated using overall accuracy, category-specific sensitivity and specificity, and the Brier score.

For LOS, a quasi-Poisson regression model was used to account for overdispersion (*Text S3F*). Pooled coefficients from the training imputation data sets defined the prediction model, which was then applied to the validation sample. Model performance was assessed using mean squared error and the mean prediction error (the mean difference between predicted and observed LOS).

Development and validation of the OTOP model were conducted and reported in accordance with the TRIPOD guidelines^13^; a completed TRIPOD checklist is provided in *Table S2*.

### Ethical considerations

Ethics approval for the use of deidentified trauma information was obtained from the Health Research and Ethics Committee, Western Australia (RGS0000004709).

## Results

### Cohort characteristics

This study analysed 105 972 trauma admissions of patients aged ≥ 50 years between 2000 and 2020 (84 778 (80%) patients in the training cohort, 21 194 (20%) in the validation cohort). The mean(standard deviation (s.d.)) age of the overall population was 74.0(13.3) years, and 49 973 (45.3%) were male. Full details of predictor variables, including the proportion of missing data, are presented in *Table 1*. A summary of outcomes examined, in-hospital mortality, surgical intervention, ICU admission, medical complications, discharge destination, and LOS is also provided in *Table 1*.

### Model development and validation

**Fig. 1:**
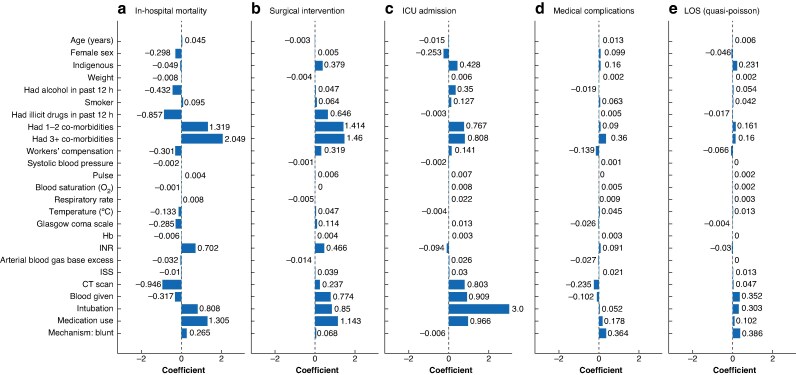
Final model coefficients for logistic regression models **a** In-hospital mortality, **b** surgical intervention, **c** ICU admission, **d** medical complications, and **e** LOS (quasi-Poisson). ICU, intensive care unit; LOS, length of hospital stay; h, hours; min, minutes.

**Fig. 2:**
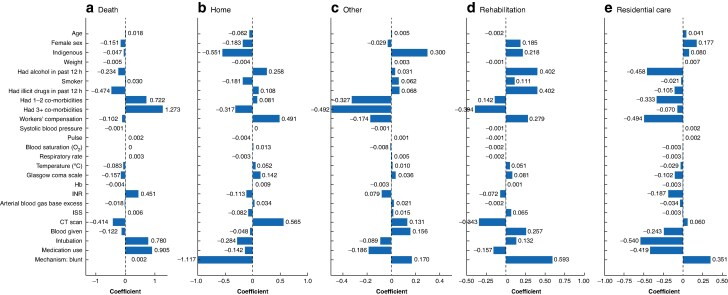
Model coefficients for discharge destinations **a** Death, **b** home, **c** other, **d** rehabilitation, and **e** residential care. h, hours; SBP, systolic blood pressure; O_2_, oxygen; Hb, haemoglobin; INR, international normalized ratio of prothrombin time; ISS, inury severity score; CT, computed tomography.

**Fig. 3:**
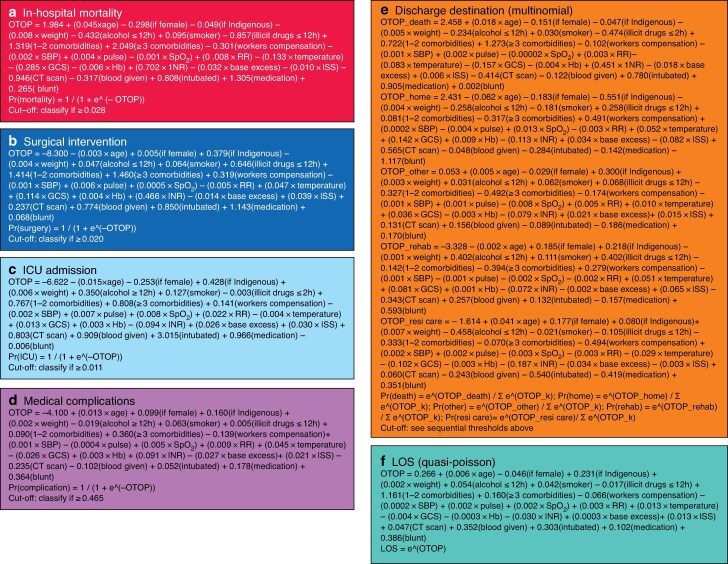
Outcome model development Logistic regression models for **a** in-hospital mortality, **b** surgical intervention, **c** ICU admission, **d** medical complications, **e** multinomial regression models for discharge destination, and **f** Poisson regression models for LOS. ICU, intensive care unit; LOS, length of hospital stay; OTOP, older trauma outcome predictor; h, hours; SBP, Systolic blood pressure; *S*_p_O_2_, peripheral oxygen saturation; RR, respiration rate; GCS, Glasgow Coma Scale; Hb, haemoglobin; INR, international normalized ratio of prothrombin time; ISS, injury severity score; CT, computed tomography; blunt, blunt injury.

**Fig. 4:**
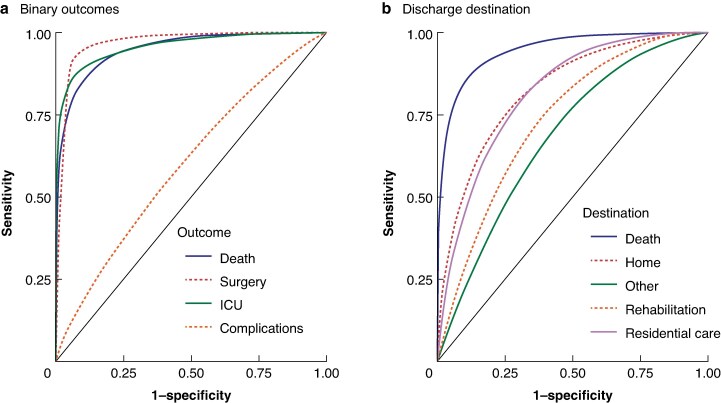
Model performance assessed using areas under receiver operating characteristic curves ICU, intensive care unit.

Predictive coefficients derived from the training cohort are shown in *Figs 1* and *2*. Predicted risks were converted to outcome probabilities, with optimal thresholds identified for clinical risk classification (*Fig. 3*). Model performance was evaluated using discrimination (AUC), calibration (Brier score), and accuracy metrics, as shown in *Fig. 4*.

#### In-hospital mortality

The model coefficients for predicting in-hospital mortality, identified from the training sample, are shown in *Fig. 1a*. Notable variables that increase the risk of the highest positive coefficient for in-hospital mortality are co-morbidities, especially the presence of more than three morbidities (coefficients of 1.319 and 2.049, respectively). Female sex, being Indigenous, and increased weight had a negative effect on inpatient death after trauma.

The final prediction model for in-hospital mortality is presented in *Fig. 3a* and *Text S1A*. The optimal cut-off for predicted probabilities was 0.028.

In the validation samples, in the final model, the mean(s.d.) AUC was 0.95(0.004), accuracy was 88.0%(3.2), sensitivity was 86.6%(2.9), specificity was 88.0%(3.4), and Brier score was 0.140(0.03), as shown in *Fig. 4a*. In comparison, the GTOS model applied to the validation data sets yielded a mean(s.d.) AUC of 0.83(0.000) and a Brier Score of 0.0230(< 0.000).

#### Surgical intervention

The co-morbidity burden again had notable coefficients for surgical interventions (1.414 for one to two co-morbidities, 1.460 for three or more co-morbidities; *Fig. 1b*). Blood transfusion, intubation, computed tomography scans, and medication requirements had positive coefficients (0.774, 0.85, and 1.14, respectively). In contrast, age, weight, systolic blood pressure, and respiratory rate were associated with weak negative coefficients (−0.003, −0.004, −0.001, and −0.005, respectively) for surgical interventions.

The final prediction model is shown in *Fig. 3b*, with details provided in *Text S1B*. The optimal cut-off for predicted probabilities was 0.020.

In the validation data sets (*Fig. 4a*), the model achieved a mean(s.d.) AUC of 0.97(0.004), accuracy of 88.8%(3.0), sensitivity of 95.1%(0.9), specificity of 88.5%(3.1), and Brier score of 0.148 (0.030).

#### ICU admissions

Model coefficients for ICU admissions are presented in *Fig. 1c*. Intubation after trauma showed remarkable coefficients for ICU admission (3.0), followed by blood transfusion and computed tomography scans (0.909 and 0.803, respectively).

The final prediction model is presented in *Fig. 3c* and *Text S1C*. The optimal cut-off for predicted probabilities was 0.011.

Running the model on the validation data (*Fig. 4a*) produced a mean(s.d.) AUC of 0.96(0.004), accuracy of 91.0%(1.9), sensitivity of 88.2%(1.2), specificity of 91.1%(1.9), and Brier score of 0.104(0.019).

#### Medical complications

The main positive coefficient was noted for blunt compared with penetrating injuries (0.364; *Fig. 1d*).

The final prediction model is presented in *Fig. 3d* and *Text S1D*, and the optimal cut-off for predicted probabilities was 0.465. Model performance was modest, with a mean(s.d.) AUC of 0.60(0.026), accuracy of 56.5%(4.0), sensitivity of 57.4%(16.4), specificity of 55.3%(16.8), and Brier score of 0.503(0.167) from the validation data sets (*Fig. 4a*).

#### Hospital length of stay

The model coefficients for LOS are presented in *Fig. 1e*. Coefficients for LOS prediction were small, the prominent being for blunt trauma (0.386). Several physiological predictors had coefficients close to zero, including systolic blood pressure, haemoglobin, and base excess.

The final prediction model is presented in *Fig. 3f* and *Text S3F*. Across the validation data sets, the model yielded a mean(s.d.) mean-squared error of 80.0(0.14) days and a mean absolute error of 4.86(0.09) days.

#### Discharge destination

The model coefficients for each of the five discharge destinations are shown in *Fig. 2*. Multinomial modelling identified more than three co-morbidities as a strong predictor of death (1.273), but negative coefficients for discharge to home (−0.317), other facilities (−0.492), rehabilitation (−0.394), and residential care (−0.070).

The final prediction model is presented in *Fig. 4b* and *Text S2E*. The optimal cut-offs for discharge destinations were 0.0297 for death, 0.527 for home, 0.185 for residential care, and 0.171 for rehabilitation (note, these are sequentially applied, so only probabilities that are not classified into one of the previous discharge destinations are then considered for the current cut-off). The validation data sets produced a mean(s.d.) accuracy of 49.2%(3.7) and a Brier Score of 0.544(0.008). The sensitivities and specificities for each of the five discharge destination outcomes are presented in *Table 2*.

**Table 2:** Mean sensitivities and specificities for each discharge destination predicted in the validation sample

Discharge destination	Sensitivity (%)		Specificity (%)	
	Mean(s.d.)	Range	Mean(s.d.)	Range
Death	87.5(3.0)	79.1–94.0	86.4(4.0)	73.4–92.9
Home	71.1(7.3)	49.4–85.4	77.3(6.4)	60.1–91.3
Other	17.7(5.8)	7.3–33.0	88.4(3.3)	79.1–96.0
Rehabilitation	26.4(4.5)	16.5–36.5	89.5(2.0)	83.5–93.9
Residential care	31.8(8.0)	9.7–46.8	90.7(4.2)	79.0–98.6

## Discussion

This is the first multi-outcome prognostic model for older adults after trauma. Unlike existing tools that focus solely on mortality, the OTOP model computes individual probabilities of six clinically relevant outcomes, including ICU admission, surgical intervention, LOS, and discharge destination.

In mortality prediction, OTOP outperformed all established scores regarding the AUC. The widely used GTOS, which was introduced in 2015, is based on age, injury severity, and the need for transfusion. It achieved an AUC of 0.83 in external validation^6^, consistent with the findings of the present study. The Elderly Mortality After Trauma score^7^, developed in 2020 with both quick and full versions, reported AUCs of 0.84–0.86, whereas the more recent Geriatric Trauma Mortality Score model (2021)^8^ achieved an AUC of 0.80. In contrast, the OTOP reached an AUC of 0.95 in validation, indicating superior discrimination. Furthermore, the application of outcome-specific cut-offs enables clear stratification of patients into high- and low-risk groups at admission, which none of the earlier models provided.

The extension of prognostic modelling beyond mortality is a central strength of the OTOP model. Predictive accuracy was excellent for ICU admission and surgical intervention, moderate for LOS, and limited for medical complications. Medical complications are highly variable, and this limitation is expected. However, by incorporating multiple endpoints, OTOP enables clinicians to estimate individualized risks across the inpatient stay, allowing early prognostication, targeted care planning, and more efficient resource allocation.

The practical application of OTOP will require digital implementation, because the model’s complexity precludes bedside calculation. An online or embedded clinical tool could automatically generate probability estimates and risk classifications. In practice, low-risk patients could proceed along standard trauma pathways. In contrast, high-risk patients may benefit from a structured multidisciplinary approach and a comprehensive geriatric assessment, ensuring closer monitoring, anticipatory management, and timelier referral to rehabilitation or residential care services.

The present study has limitations. The model was developed and validated using data from a single statewide trauma registry, and external validation in other populations is needed. A key limitation of this study is substantial missing data across several registry variables, reflecting the realities of retrospective data collection over an extended period. Multiple imputation was therefore used to reduce bias and maximize the use of available information. Under this approach, calibration should ideally be assessed within each imputed data set and then summarized across imputations. However, the large number of imputed data sets required to address missingness made the generation and presentation of calibration plots impractical in this phase of the study. Instead, overall model performance was assessed using threshold-independent measures, such as the AUC and Brier score, which enabled robust evaluation while maintaining model simplicity. The performance in predicting medical complications was modest, reflecting either limitations of registry coding or the inherent unpredictability and variability of complications. Despite these limitations, OTOP represents a major advance by providing robust, multi-outcome prognostication for older trauma patients.

Although simplified models are often feasible for single-outcome prediction, achieving similar parsimony in a multi-outcome framework is inherently more challenging. Increasing model complexity may improve discrimination but can compromise usability, particularly in emergency settings where rapid decision-making is required. In developing OTOP, the aim was therefore to balance simplicity and predictive breadth, retaining variables in straightforward formats and limiting complexity wherever possible. This approach allowed simultaneous prediction of multiple clinically relevant outcomes while preserving practical applicability at the point of care.

In summary, OTOP is the first prognostic model to deliver accurate, multi-outcome prediction for older adults after trauma. By extending prognostication beyond mortality to include intensive care, surgery, discharge, and LOS, OTOP provides a framework for early risk stratification and personalized care. Its integration into digital tools could support multidisciplinary decision-making, optimize resource allocation, and improve outcomes in this growing patient population. Further studies may be necessary to validate the model in other communities and healthcare systems.

## Supplementary Material

zrag084_Supplementary_Data

## Data Availability

The data used in this study were obtained from the Western Australia Trauma Registry and the Western Australia Data Linkage System. These data sets contain identifiable health information and cannot be made publicly available due to legal, ethical, and privacy restrictions under Western Australian legislation and data governance policies. Access to these data requires approval from the relevant data custodians and the Western Australian Department of Health Human Research Ethics Committee. Researchers wishing to access the data may submit a request through the Western Australia Data Linkage Branch, subject to institutional ethics approval and data custodian permissions.
