# Supplementary material for: Multi-outcome prognostic modelling for older adults after trauma: development and validation of the Older Trauma Outcome Predictor (OTOP) model
Source: BJS Open. 2026 Jul 20;10(4):zrag084. doi: 10.1093/bjsopen/zrag084 (PMC13381784; doi:10.1093/bjsopen/zrag084)
Supplement: zrag084_Supplementary_Data [file zrag084_supplementary_data.docx]

Title: Multi-outcome prognostic modelling for older adults after trauma: *development and validation of the Older Trauma Outcome Predictor (OTOP) model.*

Authors

Mayura Iddagoda^1,2^ , Michelle Trevenen^1^, Dieter G. Weber^3,4^, Christopher Etherton-Beer^1,2^ , Leon Flicker^1,2^

1. School of Medicine, University of Western Australia, Perth, Australia.
2. Perioperative and aged care Service, Royal Perth Hospital, Perth, Australia.
3. State Adult Major Trauma Service, Royal Perth Hospital, Perth, Australia.
4. School of Surgery, University of Western Australia, Perth, Australia

**Corresponding author**

**ORCID : 0000-0002-0631-5797**

Dr Mayura Iddagoda, MMed, FRACP

University of Western Australia, 35 Stirling Highway, Crawley WA 6009, Australia

Email: [Mayura.Iddagoda@health.wa.gov.au](mailto:Mayura.Iddagoda@health.wa.gov.au), Mayura.Iddagoda@uwa.edu.au

**SUPPLEMENTARY Appendix - Index**

Contents

[Supplementary Appendix 1 Auxiliary variables 2](#_Toc222219653)

[Table S1 Summary table of auxiliary variables for the study population 2](#_Toc222219654)

[Supplementary Appendix 2 OTOP model development statistics for each outcome 3](#_Toc222219655)

[Text S1 Logistic regression models 3](#_Toc222219656)

[Text S2 Multinomial logistic regression model 7](#_Toc222219657)

[Text S3 Quasi-Poisson regression model 10](#_Toc222219658)

[Supplementary Appendix 3 TRIPOD checklist 12](#_Toc222219659)

Table S2 [TRIPOD Checklist for the Older Trauma Outcome Predictor (OTOP) 12](#_Toc222219660)

# Supplementary Appendix 1 Auxiliary variables

## S1 Summary table of auxiliary variables for the study population

| Auxiliary variables | **Training Group** | **Validation Group** | **Overall** |
| --- | --- | --- | --- |
|  | **(N=84778)** | **(N=21194)** | **(N=105972)** |
| Trauma type |  |  |  |
| Major | 7583 (8.9%) | 1889 (8.9%) | 9472 (8.9%) |
| Minor | 77195 (91.1%) | 19305 (91.1%) | 96500 (91.1%) |
| Admission Triage |  |  |  |
| Emergency | 12421 (14.7%) | 3028 (14.3%) | 15449 (14.6%) |
| Resus | 3720 (4.4%) | 930 (4.4%) | 4650 (4.4%) |
| Semi-urgent | 20305 (24.0%) | 5074 (23.9%) | 25379 (23.9%) |
| Urgent | 38934 (45.9%) | 9739 (46.0%) | 48673 (45.9%) |
| Missing | 9398 (11.1%) | 2423 (11.4%) | 11821 (11.2%) |
| Admitted to STU |  |  |  |
| No | 82134 (96.9%) | 20524 (96.8%) | 102658 (96.9%) |
| Yes | 2644 (3.1%) | 670 (3.2%) | 3314 (3.1%) |

# Supplementary Appendix 2 ETOP model development statistics for each outcome

Text S1 ***Logistic regression models***

*S1A – In-hospital mortality*

*S1B – Surgical intervention*

*S1C – ICU admissions*

*S1D – Medical Complications*

***Text S2*** ***Multinomial logistic regression model***

*S2E – Discharge destination*

***Text S3 Quasi-Poisson regression model***

*S3F – Hospital length of stay*

## Text S1 Logistic regression models

This section describes the binomial models and how the predicted probabilities are calculated. The cut-point represents a value where any predicted probability above that value is categorised as a ‘yes’ outcome, whereas any predicted probability below the cut-point is categorised as a ‘no’ outcome. The optimal cut-point was identified by maximising the sum of the sensitivity (the number of ‘yes’ outcomes correctly classified by the model divided by the total number of ‘yes’ outcomes multiplied by 100) and specificity (the number of ‘no’ outcomes correctly classified by the model divided by the total number of ‘no outcomes multiplied by 100), and these optimal cut-points were pooled across imputation datasets to define the optimal cut-point for the prediction model. The coefficients from each of the training imputation datasets was pooled to create the final prediction model. This prediction model, with the optimal cut-point, was then applied to each of the 100 imputation datasets for patients in the validation group, and model performance measures of the area under the receiver operator characteristic curve (AUROC), accuracy (total number of correctly identified outcome events divided by the total number of outcome events multiplied by 100), sensitivity, specificity and the Brier Score were calculated and are reported. For in-hospital death, we also ran the existing GTOS prediction model on our validation dataset to compare model performance of this to ours.

Let the binary response variable take on values in $G=\{0, 1\}$. The predicted probability of outcome event equal to $1$ is of the form:

$$\Pr\left( G=1 | X=x \right)= \frac{1}{1+ e^{-(\beta_{0}+ \beta_{1}x_{1} + \ldots+ \beta_{k}x_{k} )}}$$

Where $\beta_{0}$ represents the intercept, $x_{1}, \ldots,x_{k}$ represent the $k$ predictor variables and $\beta_{1}, \ldots, \beta_{k}$ represent the model coefficients for the $k$ predictor variables. For ease of representing the final prediction models below, let:

$${OTOP= \beta}_{0}+ \beta_{1}x_{1} + \ldots+ \beta_{k}x_{k}$$

Thus,

$$\Pr\left( G=1 | X=x \right)= \frac{1}{1+ e^{-(OTOP)}}$$

*S1A – In-hospital mortality*

The predicted probability of in-hospital death can be calculated for a specific set of predictors variable values using the coefficients from Table 3 and the following two steps. Firstly,

$$OTOP=1.984+\left( 0.045*age \right)-0.298\left[ if female gender \right]-0.049\left[ if indigenous \right]-\left( 0.008*weight in kg \right)-0.432\left[ if had alcohol in last 12 hours \right]+0.095\left[ if smoker \right]-0.857\left[ if had illicit drugs in last 12 hours \right]+1.319\left[ if had 1 or 2 comorbidities \right]+2.049\left[ if had \geq3 comorbidities \right]-0.301\left[ if workers compensation \right]-\left( 0.002* systolic blood pressure \right)+\left( 0.004*pulse \right)-\left( 0.001*blood saturation \right)+\left( 0.008*respiratory rate \right)-\left( 0.133*temperature \right)-\left( 0.285*Glasgow Coma Scale \right)-\left( 0.006*Hb \right)+\left( 0.702*INR \right)-\left( 0.032*arterial blood gas base excess \right)-\left( 0.010*ISS \right)-0.946\left[ if had CT scan \right]-0.317\left[ if blood given \right]+0.808\left[ if intubated \right]+1.305\left[ if using medication \right]+0.265[if mechanism blunt]$$

This can then be used to calculate the predicted probability of in-hospital mortality with the following formula:

$\Pr($in-hospital mortality$)= \frac{1}{1+ e^{-(OTOP)}}$

If this predicted probability is greater than or equal to 0.028 then the predicted outcome is in-hospital death, whereas if the predicted probability is lower than 0.028 then the predicted outcome is no in-hospital death.

*S1B – Surgical intervention*

The predicted probability of having surgery can be calculated for a specific set of predictors variable values using the coefficients from Table 3 and the following two steps. Firstly,

$$OTOP=-8.3-\left( 0.003*age \right)+0.005\left[ if female gender \right]+0.379\left[ if indigenous \right]-\left( 0.004*weight in kg \right)+0.047\left[ if had alcohol in last 12 hours \right]+0.064\left[ if smoker \right]+0.646\left[ if had illicit drugs in last 12 hours \right]+1.414\left[ if had 1 or 2 comorbidities \right]+1.460\left[ if had \geq3 comorbidities \right]+0.319\left[ if workers compensation \right]-\left( 0.001* systolic blood pressure \right)+\left( 0.006*pulse \right)+\left( 0.0005*blood saturation \right)-\left( 0.005*respiratory rate \right)+\left( 0.047*temperature \right)+\left( 0.114*Glasgow Coma Scale \right)+\left( 0.004*Hb \right)+\left( 0.466*INR \right)-\left( 0.014*arterial blood gas base excess \right)+\left( 0.039*ISS \right)+0.237\left[ if had CT scan \right]+0.774\left[ if blood given \right]+0.850\left[ if intubated \right]+1.143\left[ if using medication \right]+0.068[if mechanism blunt]$$

This can then be used to calculate the predicted probability of having surgery with the following formula:

$\Pr($surgery$)= \frac{1}{1+ e^{-(OTOP)}}$

If this predicted probability is greater than or equal to 0.020 then the predicted outcome is having surgery, whereas if the predicted probability is lower than 0.020 then the predicted outcome is not having surgery.

*S1C – ICU admissions*

The predicted probability of being admitted to ICU can be calculated for a specific set of predictors variable values using the coefficients from Table 3 and the following two steps. Firstly,

$$OTOP=-6.622-\left( 0.015*age \right)-0.253\left[ if female gender \right]+0.428\left[ if indigenous \right]+\left( 0.006*weight in kg \right)+0.350\left[ if had alcohol in last 12 hours \right]+0.127\left[ if smoker \right]-0.003\left[ if had illicit drugs in last 12 hours \right]+0.767\left[ if had 1 or 2 comorbidities \right]+0.808\left[ if had \geq3 comorbidities \right]+0.141\left[ if workers compensation \right]-\left( 0.002* systolic blood pressure \right)+\left( 0.007*pulse \right)+\left( 0.008*blood saturation \right)+\left( 0.022*respiratory rate \right)-\left( 0.004*temperature \right)+\left( 0.013*Glasgow Coma Scale \right)+\left( 0.003*Hb \right)-\left( 0.094*INR \right)+\left( 0.026*arterial blood gas base excess \right)+\left( 0.030*ISS \right)+0.803\left[ if had CT scan \right]+0.909\left[ if blood given \right]+3.015\left[ if intubated \right]+0.966\left[ if using medication \right]-0.006[if mechanism blunt]$$

This can then be used to calculate the predicted probability of being admitted to ICY with the following formula:

$\Pr($ICU admission$)= \frac{1}{1+ e^{-(OTOP)}}$

If this predicted probability is greater than or equal to 0.011 then the predicted outcome is being admitted to ICU, whereas if the predicted probability is lower than 0.011 then the predicted outcome is not being admitted to ICU.

*S1D – Medical Complications*

The predicted probability of having medical complications can be calculated for a specific set of predictors variable values using the coefficients from Table 3 and the following two steps. Firstly,

$$OTOP=-4.1+\left( 0.013*age \right)+0.099\left[ if female gender \right]+0.160\left[ if indigenous \right]+\left( 0.002*weight in kg \right)-0.019\left[ if had alcohol in last 12 hours \right]+0.063\left[ if smoker \right]+0.005\left[ if had illicit drugs in last 12 hours \right]+0.090\left[ if had 1 or 2 comorbidities \right]+0.360\left[ if had \geq3 comorbidities \right]-0.139\left[ if workers compensation \right]+\left( 0.001* systolic blood pressure \right)-\left( 0.0004*pulse \right)+\left( 0.005*blood saturation \right)+\left( 0.009*respiratory rate \right)+\left( 0.045*temperature \right)-\left( 0.026*Glasgow Coma Scale \right)+\left( 0.003*Hb \right)+\left( 0.091*INR \right)-\left( 0.027*arterial blood gas base excess \right)+\left( 0.021*ISS \right)-0.235\left[ if had CT scan \right]-0.102\left[ if blood given \right]+0.052\left[ if intubated \right]+0.178\left[ if using medication \right]+0.364[if mechanism blunt]$$

This can then be used to calculate the predicted probability of having medical complications with the following formula:

$\Pr($medical complication$)= \frac{1}{1+ e^{-(OTOP)}}$

If this predicted probability is greater than or equal to 0.465 then the predicted outcome is having medical complications, whereas if the predicted probability is lower than 0.465 then the predicted outcome is not having medical complication

## Text S2 Multinomial logistic regression model

For the multinomial logistic regression modelling discharge destination, the predicted probabilities for each of the five discharge destinations, for each imputation dataset for patients in the training group were calculated. Appendix B describes the how the predicted probabilities are calculated in a multinomial logistic regression model. We considered sequential optimal cut-points in order to assign predicted probabilities to discharge destination classes. Specifically, the optimal cut-point was first derived, using the same technique as detailed for the binary outcomes above, for those with a discharge destination of death. Then an optimal cut-point was identified for the discharge destination of home, considering only the patients who did not have a discharge destination of death. Next, only patients who were not already classified with a discharge destination of either death or home, were used to identify the optimal cut-point for a discharge destination of residential care. Finally, for those not already assigned to a discharge destination of death, home or residential care, the optimal cut-point was identified on predicted probabilities for rehabilitation, where those who were not categorised into the discharge destination of rehabilitation were assigned the discharge destination of ‘other’. The coefficients from the imputation datasets of the training group were pooled to create the coefficients in the final prediction model. This prediction model, with the optimal sequential cut-points, was then applied to the imputation datasets of those in the validation group. Model performance measures of overall accuracy, sensitivity and specificity of each discharge destination and the Brier Score were calculated and are reported.

Let the multinomial response variables have $J$ class $G=\{1, 2, .. ., J\}$. The predicted probability of outcome $j$ is of the form:

$$\Pr\left( G=j | X=x \right)= \frac{e^{\beta_{0j} + \beta_{1j}x_{1} + \ldots+\beta_{kj}x_{k}}}{\sum_{c=1}^{J} e^{\beta_{0c} + \beta_{1c}x_{1} + \ldots+\beta_{kc}x_{k}}}$$

Where $\beta_{0c}$ represents the intercept for response level $j$, $x_{1}, \ldots,x_{k}$ represent the $k$ predictor variables and $\beta_{1c}, \ldots, \beta_{kc}$ represent the model coefficients of the $k$ predictor variables for response level $c$. For ease of representing the final prediction models below, let:

$${OTOP}_{c}= \beta_{0c}+ \beta_{1c}x_{1}+\ldots+ \beta_{kc}x_{k}$$

*S2E – Discharge destination*

The predicted probabilities for each class of the discharge destination outcome can be calculated using the coefficients in Table 4 and following the following steps. Firstly, calculate ${resp}_{c}$ for each outcome class: death, home, other, rehabilitation and residential care.

$${OTOP}_{death}=2.458+\left( 0.018*age \right)-0.151\left[ if female gender \right]-0.047\left[ if indigenous \right]-\left( 0.005*weight in kg \right)-0.234\left[ if had alcohol in last 12 hours \right]+0.030\left[ if smoker \right]-0.474\left[ if had illicit drugs in last 12 hours \right]+0.722\left[ if had 1 or 2 comorbidities \right]+1.273\left[ if had \geq3 comorbidities \right]-0.102\left[ if workers compensation \right]-\left( 0.001* systolic blood pressure \right)+\left( 0.002*pulse \right)-\left( 0.00002*blood saturation \right)+\left( 0.003*respiratory rate \right)-\left( 0.083*temperature \right)-\left( 0.157*Glasgow Coma Scale \right)-\left( 0.004*Hb \right)+\left( 0.451*INR \right)-\left( 0.018*arterial blood gas base excess \right)+\left( 0.006*ISS \right)-0.414\left[ if had CT scan \right]-0.122\left[ if blood given \right]+0.780\left[ if intubated \right]+0.905\left[ if using medication \right]+0.002[if mechanism blunt]$$

$${OTOP}_{home}=2.431-\left( 0.062*age \right)-0.183\left[ if female gender \right]-0.551\left[ if indigenous \right]-\left( 0.004*weight in kg \right)-0.258\left[ if had alcohol in last 12 hours \right]-0.181\left[ if smoker \right]+0.258\left[ if had illicit drugs in last 12 hours \right]+0.081\left[ if had 1 or 2 comorbidities \right]-0.317\left[ if had \geq3 comorbidities \right]+0.491\left[ if workers compensation \right]+\left( 0.0002* systolic blood pressure \right)-\left( 0.004*pulse \right)+\left( 0.013*blood saturation \right)-\left( 0.003*respiratory rate \right)+\left( 0.052*temperature \right)+\left( 0.142*Glasgow Coma Scale \right)+\left( 0.009*Hb \right)-\left( 0.113*INR \right)+\left( 0.034*arterial blood gas base excess \right)-\left( 0.082*ISS \right)+0.565\left[ if had CT scan \right]-0.048\left[ if blood given \right]-0.284\left[ if intubated \right]-0.142\left[ if using medication \right]-1.117[if mechanism blunt]$$

$${OTOP}_{other}=0.053+\left( 0.005*age \right)-0.029\left[ if female gender \right]+0.300\left[ if indigenous \right]+\left( 0.003*weight in kg \right)+0.031\left[ if had alcohol in last 12 hours \right]+0.062\left[ if smoker \right]+0.068\left[ if had illicit drugs in last 12 hours \right]-0.327\left[ if had 1 or 2 comorbidities \right]-0.492\left[ if had \geq3 comorbidities \right]-0.174\left[ if workers compensation \right]-\left( 0.001* systolic blood pressure \right)+\left( 0.001*pulse \right)-\left( 0.008*blood saturation \right)+\left( 0.005*respiratory rate \right)+\left( 0.010*temperature \right)+\left( 0.036*Glasgow Coma Scale \right)-\left( 0.003*Hb \right)-\left( 0.079*INR \right)+\left( 0.021*arterial blood gas base excess \right)+\left( 0.015*ISS \right)+0.131\left[ if had CT scan \right]+0.156\left[ if blood given \right]-0.089\left[ if intubated \right]-0.186\left[ if using medication \right]+0.170[if mechanism blunt]$$

$${OTOP}_{rehab}=-3.328-\left( 0.002*age \right)+0.185\left[ if female gender \right]+0.218\left[ if indigenous \right]-\left( 0.001*weight in kg \right)+0.402\left[ if had alcohol in last 12 hours \right]+0.111\left[ if smoker \right]+0.402\left[ if had illicit drugs in last 12 hours \right]-0.142\left[ if had 1 or 2 comorbidities \right]-0.394\left[ if had \geq3 comorbidities \right]+0.279\left[ if workers compensation \right]-\left( 0.001* systolic blood pressure \right)-\left( 0.001*pulse \right)-\left( 0.002*blood saturation \right)-\left( 0.002*respiratory rate \right)+\left( 0.051*temperature \right)+\left( 0.081*Glasgow Coma Scale \right)+\left( 0.001*Hb \right)-\left( 0.072*INR \right)-\left( 0.002*arterial blood gas base excess \right)+\left( 0.065*ISS \right)-0.343\left[ if had CT scan \right]+0.257\left[ if blood given \right]+0.132\left[ if intubated \right]-0.157\left[ if using medication \right]+0.593[if mechanism blunt]$$

$${OTOP}_{resi care}=-1.614+\left( 0.041*age \right)+0.177\left[ if female gender \right]+0.080\left[ if indigenous \right]+\left( 0.007*weight in kg \right)-0.458\left[ if had alcohol in last 12 hours \right]-0.021\left[ if smoker \right]-0.105\left[ if had illicit drugs in last 12 hours \right]-0.333\left[ if had 1 or 2 comorbidities \right]-0.070\left[ if had \geq3 comorbidities \right]-0.494\left[ if workers compensation \right]+\left( 0.002* systolic blood pressure \right)+\left( 0.002*pulse \right)-\left( 0.003*blood saturation \right)-\left( 0.003*respiratory rate \right)-\left( 0.029*temperature \right)-\left( 0.102*Glasgow Coma Scale \right)-\left( 0.003*Hb \right)-\left( 0.187*INR \right)-\left( 0.034*arterial blood gas base excess \right)-\left( 0.003*ISS \right)+0.060\left[ if had CT scan \right]-0.243\left[ if blood given \right]-0.540\left[ if intubated \right]-0.419\left[ if using medication \right]+0.351[if mechanism blunt]$$

The above can then be used to calculate the predicted probability for each outcome class using the following formulas:

$$\Pr\left( death \right)= \frac{e^{{OTOP}_{death}}}{e^{{OTOP}_{death}}+e^{{OTOP}_{home}}+e^{{OTOP}_{other}}+e^{{OTOP}_{rehab}}+e^{{OTOP}_{resi care}}}$$

$$\Pr\left( home \right)= \frac{e^{{OTOP}_{home}}}{e^{{OTOP}_{death}}+e^{{OTOP}_{home}}+e^{{OTOP}_{other}}+e^{{OTOP}_{rehab}}+e^{{OTOP}_{resi care}}}$$

$$\Pr\left( other \right)= \frac{e^{{OTOP}_{other}}}{e^{{OTOP}_{death}}+e^{{OTOP}_{home}}+e^{{OTOP}_{other}}+e^{{OTOP}_{rehab}}+e^{{OTOP}_{resi care}}}$$

$$\Pr\left( rehab \right)= \frac{e^{{OTOP}_{rehab}}}{e^{{OTOP}_{death}}+e^{{OTOP}_{home}}+e^{{OTOP}_{other}}+e^{{OTOP}_{rehab}}+e^{{OTOP}_{resi care}}}$$

$$\Pr\left( resi care \right)= \frac{e^{{OTOP}_{resi care}}}{e^{{OTOP}_{death}}+e^{{OTOP}_{home}}+e^{{OTOP}_{other}}+e^{{OTOP}_{rehab}}+e^{{OTOP}_{resi care}}}$$

We can then use the sequential cut-offs to classify the predicted probabilities into an outcome class. Specifically, if the predicted probability of death is greater than or equal to 0.0297 then the predicted discharge destination is death. For those not classified with a discharge destination of death, if the predicted probability of home is greater than or equal to 0.527 then the predicted discharge destination is home. For those not classified with a discharge destination of death nor home, if the predicted probability for residential care is greater than or equal to 0.185 then the predicted discharge destination is residential care. For those not already classified with a discharge destination of death, home or residential care, if the predicted probability of rehabilitation is greater than or equal to 0.171 then the predicted discharge destination is rehabilitation, otherwise it is other.

## Text S3 Quasi-Poisson regression model

For the quasi-Poisson regression modelling hospital length of stay, the coefficients from the imputation datasets of the training groups were pooled to generate the coefficients for the final prediction model. Appendix C describes this model in greater detail. This model was then applied to the imputation datasets of the validation groups and model performance measures of mean-squared-error as well as the average error (i.e. difference between the predicted and observed length of stays) are reported.

The predicted outcome, $Y$, from a quasi-Poisson model may be calculated from the predictor variables and coefficients using the following equation:

$$Y= e^{\beta_{0} + \beta_{1}x_{1}+\ldots+ \beta_{k}x_{k}}$$

Where $\beta_{0}$ represents the intercept, $x_{1}, \ldots,x_{k}$ represent the $k$ predictor variables and $\beta_{1}, \ldots, \beta_{k}$ represent the model coefficients for the $k$ predictor variables. For ease of representing the final prediction models below, let:

$${OTOP= \beta}_{0}+ \beta_{1}x_{1} + \ldots+ \beta_{k}x_{k}$$

Thus,

$$Y= e^{resp}$$

*S3F – Hospital length of stay*

Using the coefficients in Table 3, the predicted length of hospital stay in days may be estimated by first calculating:

$$resp=0.266+\left( 0.006*age \right)-0.046\left[ if female gender \right]+0.231\left[ if indigenous \right]+\left( 0.002*weight in kg \right)+0.054\left[ if had alcohol in last 12 hours \right]+0.042\left[ if smoker \right]-0.017\left[ if had illicit drugs in last 12 hours \right]+1.161\left[ if had 1 or 2 comorbidities \right]+0.160\left[ if had \geq3 comorbidities \right]-0.066\left[ if workers compensation \right]-\left( 0.0002* systolic blood pressure \right)+\left( 0.002*pulse \right)+\left( 0.002*blood saturation \right)+\left( 0.003*respiratory rate \right)+\left( 0.013*temperature \right)-\left( 0.004*Glasgow Coma Scale \right)-\left( 0.0003*Hb \right)-\left( 0.030*INR \right)+\left( 0.0003*arterial blood gas base excess \right)+\left( 0.013*ISS \right)+0.047\left[ if had CT scan \right]+0.352\left[ if blood given \right]+0.303\left[ if intubated \right]+0.102\left[ if using medication \right]+0.386[if mechanism blunt]$$

This can then be used to calculate the estimated length of hospital stay as follows:

$$LOS= e^{OTOP}$$

# Supplementary Appendix 3 TRIPOD checklist

## Table S2 TRIPOD Checklist for the Older Trauma Outcome Predictor (OTOP)

| Section / Topic | Item | TRIPOD Recommendation | Where Addressed in Thesis |
| --- | --- | --- | --- |
| Title & Abstract | 1 | Identify the study as developing and/or validating a multivariable prediction model | Chapter 6 title; Thesis abstract |
| Title & Abstract | 2 | Provide a structured summary of objectives, design, participants, predictors, outcomes, and performance | Thesis abstract; Chapter 6 summary |
| Introduction | 3 | Explain medical context and rationale for model development | Chapters 1, 4, 5; Chapter 6 Introduction |
| Methods – Data Source | 4 | Describe study design and data sources | WA Trauma Registry and Data Linkage System; Chapter 6; Appendix |
| Methods – Participants | 5 | Specify eligibility criteria and setting | Chapter 6 Methods |
| Methods – Outcome | 6 | Clearly define outcomes and their assessment | Six outcomes defined; Chapter 6; Appendix |
| Methods – Predictors | 7 | Define predictors and how they were measured | Chapter 6; Appendix |
| Methods – Sample Size | 8 | Explain how sample size was determined | n = 105,972; Chapter 6 Methods |
| Methods – Missing Data | 9 | Describe handling of missing data | Multiple imputation; Chapter 6; Appendix |
| Methods – Statistical Analysis | 10 | Describe model type, predictor handling, and validation | Regression methods; split-sample validation; Chapter 6 |
| Methods – Risk Groups | 11 | Describe creation of risk groups | Chapter 6 Results |
| Results – Participants | 12 | Describe participant flow and characteristics | Chapter 6 Results |
| Results – Model Development | 13 | Report predictor coefficients and model specification | Appendix |
| Results – Model Performance | 14 | Report model performance measures | AUC, calibration, MAE; Chapter 6 |
| Results – Model Presentation | 15 | Explain how the model can be used | Equations and online calculator; Chapter 6 |
| Discussion – Limitations | 16 | Discuss limitations of the study and model | Chapter 6 Discussion; Chapter 8 |
| Discussion – Interpretation | 17 | Provide overall interpretation of results | Chapter 6 Discussion |
| Discussion – Clinical Implications | 18 | Discuss clinical use and future research | Chapter 6 Discussion; Chapter 8 |
| Other Information | 19 | Describe availability of supplementary resources | Appendices |
| Other Information | 20 | Report funding and conflicts of interest | Thesis declarations |
